# Sensitive Aptamer SERS and RRS Assays for Trace Oxytetracycline Based on the Catalytic Amplification of CuNCs

**DOI:** 10.3390/nano11102501

**Published:** 2021-09-26

**Authors:** Shuxin Chen, Xiaowen Lv, Jifan Shen, Siqi Pan, Zhiliang Jiang, Yang Xiao, Guiqing Wen

**Affiliations:** 1 Key Laboratory of Ecology of Rare and Endangered Species and Environmental Protection (Guangxi Normal University), Ministry of Education, Guilin 541004, China; shuxinchen666@163.com (S.C.); xwlv9785178@163.com (X.L.); ddyeah88@163.com (J.S.); sqpan1130@126.com (S.P.); zljiang@gxnu.edu.cn (Z.J.); xy1169311611@163.com (Y.X.); 2 Guangxi Key Laboratory of Environmental Pollution Control Theory and Technology for Science and Education Combined with Science and Technology Innovation Base, Guilin 541004, China

**Keywords:** CuNCs, OTC, aptamer, RRS, SERS

## Abstract

A new method for the determination of oxytetracycline (OTC) has been established by coupling the catalytic amplification reaction of copper nanoclusters (CuNCs) with the aptamer reaction. CuNCs prepared by a wet chemical method have the catalytic activity for the formation of gold nanoparticles (AuNPs) resulting from a HAuCl_4_-ethanol (En) reaction. The experimental results showed that OTC aptamer (Apt) can be adsorbed on the surface of CuNCs in a non-specific way, thus inhibiting its catalytic activity. When OTC was added to the solution, the OTC-Apt complex was generated by a specific reaction, which made the CuNCs desorb and restore their catalytic activity. With the increase of OTC, the recovery of the catalytic activity of CuNCs is strengthened, the reaction speed is accelerated, and the number of AuNPs is increased. The generated AuNPs exhibited surface enhanced Raman scattering (SERS) signals at 1615 cm^−1^ in the presence of Vitoria blue 4R (VB4R) molecular probes, and a resonance Rayleigh scattering (RRS) peak at 586 nm. There is a good linear relationship between the intensities of SERS, or RRS, and OTC concentration at the range of 37.5–300 ng/L or 37.5–225 ng/L, respectively. A new SERS and RRS assay for the determination of trace OTC based on the regulation of CuNCs catalysis was established.

## 1. Introduction

Metal nanoclusters have attracted much attention because of their unique physical, chemical, and optical properties, and have become a hot research field in recent decades [1]. Significant achievements have been made in the research of gold, silver, and other noble metal nanomaterials [2]. Meanwhile, copper, which is in the same group as gold and silver, has attracted extensive attention. Compared to gold and silver, copper is more abundant, available, and relatively cheap on the planet. The preparation conditions of CuNCs are simple, and it has been proved experimentally that CuNCs have similar advantages to gold and silver nanoclusters in optical properties [3]. Therefore, the research prospect and application potentials of CuNCs are very great and are worth exploring further. The preparation of CuNCs in the laboratory is usually directly reduced by using a wet chemical method, which involves adding reductive substances to make copper ions recover in the solution to produce Cu atom particles, which are further clustered until CuNCs are obtained [4]. With the research development of CuNCs in recent years, it has been found that most CuNCs using a direct reduction method have the problems of weak fluorescence, low quantum yield, and easy aggregation. To obtain CuNCs with good stability, and a suitability for experimental use, surface ligands can be added for protection during preparation, which can control the stability and rate of nanocrystalline nucleation. In combination with different methods to improve the stability of CuNCs, more synthesis processes and methods have been experimented with, such as water-in-oil (w/o) micro emulsion strategies [5] and the Brust-Schiffrin method [6], among others. It is reported that CuNCs have a great potential in optical sensing [7], environmental monitoring [8], and medical-related applications [9,10]. Han et al. [11] found that highly photoluminescent CuNCs have been developed using 2,3,5,6-tetrafluorophenol as a reducing agent and protective ligand for the rapid, sensitive, and selective detection of histamine in foods. The detection limit was as low as 60 nmol/L and the linear range was 0.1–10 μmol/L. Deng et al. [12] reported that copper nanoparticles were used as precursors and ammonia was used as an etching agent to prepare CuNCs with a good green fluorescence. When the ammonia source was from urea and hydrolyzed under the catalysis of urease, the CuNCs could be used to detect urea. The linear range for urea detection is from 0.25 to 5 mmol/L, and the limit of detection (LOD) is 0.01 mmol/L. The above studies are mainly based on the fluorescence properties of CuNCs, while there are few studies on their scattering properties. To the best of our knowledge, there are not published studies regarding CuNCs’ catalysis of HAuCl_4_-ethanol reaction and its application to the aptamer SERS/RRS for the quantitative analysis of OTC.

Aptamer is a class of single-stranded oligonucleotides with a high affinity and specific binding to targeted target molecules, including DNA, RNA, and modified RNA [13]. SELEX is known as the systematic evolution of ligands by exponential enrichment. According to the experiment of Tuerk C et al. [14,15], the basic principle is to artificially construct a single-chain random oligonucleotide ligand library by applying the new technology of modern molecular biology. The oligonucleotide ligands, which interact with special target molecules, were retained and expanded, and then they were enriched through amplification and artificial selection. Imran Mahmood Khan [16] applied aptamer to functionalize silver nanoclusters and combined them with MoS_2_ nanosheets to prepare an aptamer-silver nanoclusters fluorescent probe capable of detecting T-2 toxin. The linear range was 0.005–500 ng/mL, and the LOD was 0.93 pg/mL. Thus, a high sensitivity, simple, rapid, and efficient mycotoxin detection method was established. Zhang et al. [17] had established a new fluorescence method for the quantitative analysis of Pb^2+^ by using silver nanoclusters functionalized aptamers, and enhanced their fluorescence after the aptamers specifically recognized Pb^2+^, with a linear range of 5–50 nmol/L and the LOD of 3 nmol/L. RRS is a kind of special elastic scattering produced by the absorption spectrum of scattering molecules located near the excitation wavelength, and its intensity is much higher than that of the traditional light scattering [18]. Liu et al. [19] first used RRS signals to study the physical and chemical differences of ion complexes formed due to electrostatic attraction, hydrophobic interaction, and charge transfer interaction between small molecules. Therefore, RRS has been widely used in the determination of polymer organic matter, the research and analysis of nanoparticles, and trace inorganic small molecules, which has become a new method with a wide range of applications, accurate and reliable, with a simple operation and a low cost. Using citric acid, thiourea, and auric chloride as precursors, Wang et al. [20] prepared gold-doped carbon dots through microwave digestion-equipment, and the acidified ammonium molybdate and gold-doped carbon dots combined formed a complex by intermolecular force, which weakened the RRS signal of the reaction system. When PO_4_^3−^ is added, it will combine with ammonium molybdate to release the gold-doped carbon dots, and the RRS signal of the system will be restored. The detection range of PO_4_^3−^ was 1.4–21 nmol/L, and the LOD was 0.60 nmol/L. Hang et al. [21] proposed a convenient and sensitive spectrophotometric method for the determination of malathion (Mala) by RRS using Erythrocyte B as a probe with a detection range of 0.012–0.8 μg/mL and LOD of 1.7 ng/mL. Nano-catalysis is a kind of important signal amplification method based on the improvement of electron transfer in oxidation-reduction reaction by the surface electrons of nanoparticles, which accelerates the indicator reaction and strengthens the signal by increasing the product, thus improving the sensitivity of the method. Combined with RRS, nano-catalysis has a broad application prospect in the field of analysis [22,23,24]. Based on the catalysis of gold-doped carbon dots on the HAuCl_4_-fructose reaction to generate gold nanoparticles, Wang et al. [22] combined the RRS effect of gold nanoparticles and the regulation of As^3+^ aptamer on nano-catalysis, and established a new RRS method of nano-catalytic amplification to determine 0.10–0.60 μg/L As^3+^. Using the catalytic effect of nano gold, which was produced through CO reducing HAuCl_4_ and the RRS effect of the product, Yao et al. established a sensitive SERS/RRS dual-function probe for the determination of 3.0–413 ng/mL CO [23]. Theoretical and experimental studies show that, in general, the smaller the size of nanoparticles, the stronger the catalytic ability. Nanoclusters are smaller than ordinary nanoparticles, and their catalytic amplification effect is better [25,26,27,28]. Therefore, it is of great innovative significance to explore new catalytic amplification reactions of CuNCs and to use them in the detection of OTC by aptamer RRS.

OTC is a tetracycline antibiotics and its molecular formula is C_22_H_24_N_2_O_9_. It is a broad-spectrum antibiotic with antibacterial activity and can be used as an additive in food. In the environment, OTC diffuses into the water environment with the discharge of tail water from sewage plants, surface runoff, and garbage exudate, thus affecting the water quality and human health. Many reports indicate that antibiotics have been detected in groundwater and water sources. OTC in the environment with contact absorption into the organism will cause antibiotic resistance in human body, thus affecting health [29]. Therefore, accurate detection of OTC content is of positive significance for environmental protection and human health. At present, the main methods for determining OTC are high performance liquid chromatography, electrochemical method, fluorescence photometry, immunoassay, and so on. But some require expensive instruments, and complex procedures or are not sensitive enough [30,31,32,33,34,35]. So, it is of great practical significance to explore a more sensitive and simple method for detecting OTC. González Fá et al. [36] prepared silver nanoparticles with glucose as a reducing agent and used them as SERS substrate to detect OTC in honey. The detection limits can be as low as 15.3 ppb. Gao et al. [37] constructed two high-efficiency fluorescent probes; they are orange peel carbon quantum dots (ON-CQDs) and watermelon peel carbon quantum dots (WN-CQDs), which are compatible with OTC concentrations at 2–100 mol/L and 0.25–100 mol/L, respectively. Moreover, the LOD were 0.973 µmol/L (ON-CQDs) and 0.077 µmol/L (WN-CQDs). In this article, a kind of high catalytic activity of CuNCs was prepared with CuSO_4_ as a precursor and L-cysteine as reducing agent in alkaline conditions. Aptamers can form complexly with it. Combining the regulation of aptamer on CuNCs catalytic effect with the scattering properties of the product of nanogold particles, a simple, sensitive, and selective method was established for the determination of OTC, which can provide a reliable basis for the determination of OTC in water samples.

## 2. Materials and Methods

### 2.1. Instruments

A model of DXR smart Raman spectrometer (Thermo, Waltham, MA, USA), with a laser wavelength of 633 nm, power of 3.5 mW, slit of 50 μm, and acquisition time of 5.0 s, a model of CaryEclipse fluorescence spectrophotometer (Hitachi Company, Tokyo, Japan), a model of TU-1901 dual-beam ultraviolet-visible spectrophotometer (Beijing General Analysis General Instrument Co., Ltd., Beijing, China), a model of 79-1 Magnetic Heating Stirrer (Jiangsu Zhongda Instrument Factory, Jiangsu, China), and a model of HH-2 digital constant temperature water bath (Changzhou Guohua Electric Appliance Co., Ltd., Changzhou, China) were used.

### 2.2. Reagents

84 μmol/L HAuCl_4_ (Sinopharmaceutical Chemical Reagents Co., Ltd., Shanghai, China), 1 mmol/L CuSO_4_, 1 mol/L NaOH, 35 mg/mL L-cysteine, 100 μmol/L oxytetracycline aptamer (Apt) with sequence of CGA CGC ACA GTC GGT GCG TAC CTG GTT GCC GTT GTG T (Shanghai Sangon Bioengineering Co., Ltd., Shanghai, China). The binding force between aptamer and target is closely related to base composition and base number. In order to select a suitable aptamer, it is necessary to refer to previous experience [33] and experimental verification. 10 μg/L oxytetracycline (OTC) solution (Sinopharmaceutical Chemical Reagent Co., Ltd., Shanghai, China), ethyl-alcohol (Guangdong Guanghua Sci-Tech Co., Ltd., Guangdong, China) and 10 mmol/L HCl (Sichuan Xilong Science Co., Ltd., Sichuan, China) were used. All reagents were analytical grade, and the experimental water was ultrapure water.

### 2.3. Preparation of Copper Nanoclusters (CuNCs)

1 mL 1 mmol/L CuSO_4_ was added drop by drop into 10 mL reductant solution [L-cysteine (35 mg/mL) + NaOH (1 mol/L)], then heated in a 60 °C water bath with stirring for 4.5 h, and a yellow clarification solution was obtained after the reaction. The solution was cooled to room temperature, and the impurities were removed with a 0.2 μm filter membrane. The volume was fixed to 25 mL, and the concentration of the solution was measured by Cu^2+^, which was 0.04 mmol/L.

### 2.4. Procedure

In a 5.0 mL stoppered test tube, 60 μL 0.04 mmol/L CuNCs and 55 μL 1 nmol/L Apt solutions were added sequentially, successively, and let stand for 5 min. Subsequently, a certain concentration of OTC, 100 μL 0.1%HAuCl_4_, 130 μL 10 mmol/L HCl, and 110 μL ethanol were added and diluted to 2 mL. After the reagents were mixed fully, they were placed in a water bath at 60 °C for static reaction, and then placed in ice water for 6 min to quickly cool to room temperature. Resonance scattering spectra were obtained by synchronous scanning with a fluorescence spectrophotometer Volt = 350 V, excited slit = emission slit = 10 nm, emission filter = 1%T attenuator, λ_ex_ − λ_em_ = ∆λ = 0. The RRS intensity at 586 nm (*I*_586 nm_) was measured. Without adding OTC to blank, to determine its blank value and calculate ∆*I*_586 nm_ = *I*_586 nm_ − *(I*_586 nm_*)*_0_. A 50 μL10 µmol/L VB4R was added for SERS detection. The SERS value at 1615 cm^−1^ was recorded as *I*_1615 cm_^−1^, the SERS value without OTC was taken as the blank value (*I*_1615 cm_^−1^)_0_, and the value of △*I*_1615 cm_^−1^ = *I*_1615 cm_^−1^ − (*I*_1615 cm_^−1^)_0_ was calculated. 

## 3. Results and Discussion

### 3.1. Analysis of the Principle

**Figure 1:**
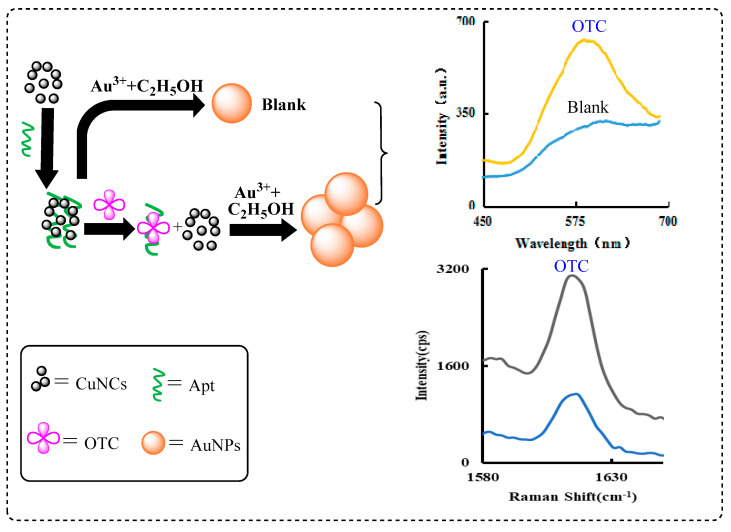
Schematic diagram of RRS/SERS assay of OTC based on Apt controlling CuNCs catalysis.

The reduction solution of L-cysteine and NaOH can reduce CuSO_4_ to produce CuNCs. The newly formed CuNCs are stable and have strong fluorescence and good catalytic activity. Under normal conditions, due to the absence of the catalyst, the reaction of HAuCl_4_-ethanol was very slow, and few AuNPs were produced, so the RRS signal value was also extremely low. However, the addition of CuNCs can greatly catalyze the reaction of HAuCl_4_-ethanol, generating more AuNPs and stronger RRS signals. When the Apt was added, it could be adsorbed to the surface of CuNCs due to electrostatic attraction and the catalytic effect was inhibited. After the addition of OTC, the Apt was desorbed from the surface of the CuNCs due to the specific binding between the OTC and Apt, and the catalytic activity of the CuNCs was restored. With the increase of the OTC concentration, more and more CuNCs were released, and the catalytic generation of the AuNPs also increased. Based on the linear relationship between the RRS/SERS signals enhancement and the OTC concentration, a new RRS/SERS method for OTC was established (Figure 1).

### 3.2. Scanning Electron Microscopy (SEM) and Transmission Electron Microscopy (TEM)

**Figure 2:**
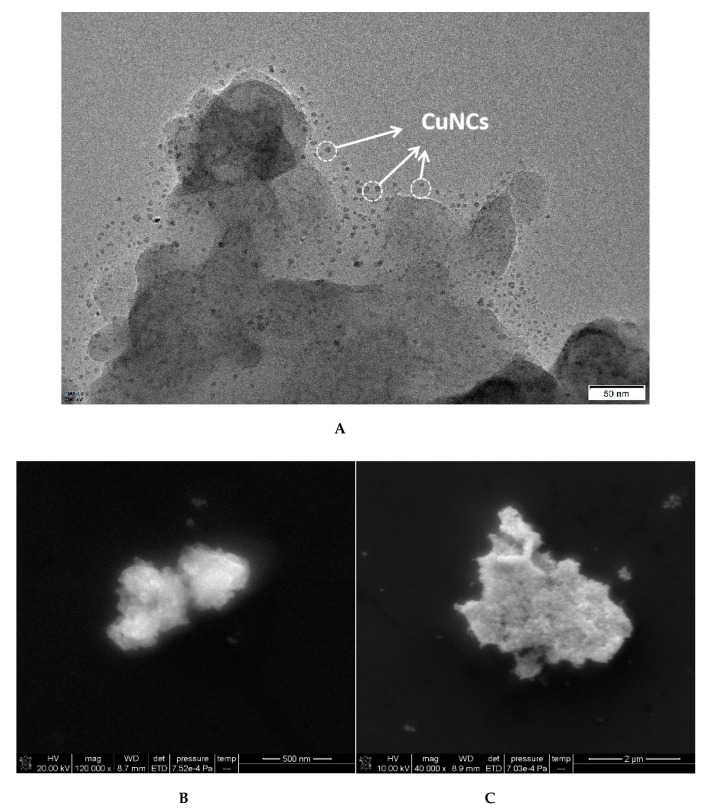
TEM and SEM of CuNCs and analysis system. (**A**) TEM of CuNCs; (**B**) SEM, 1.2 nmol/L CuNCs + 27.5 nmol/L Apt + 2.52 μmol/L HAuCl_4_ + 1.87 mol/L ethanol + 0.65 mol/L HCl + 112.5 ng/L OTC; (**C**) SEM, 1.2 nmol/L CuNCs + 27.5 nmol/L Apt + 2.52 μmol/L HAuCl_4_ + 1.87 mol/L ethanol + 0.65 mol/L HCl + 225 ng/L OTC.

The prepared CuNCs were dropped into the nickel mesh after being diluted with ethanol for a certain time and dried naturally. The samples were detected by TEM. The results show that the CuNCs particles (marked with arrows) are a uniform sphere with an average particle size of about 1–2 nm, and have a good dispersion (Figure 2A). According to the experimental method, the solution containing different concentration of OTC were centrifuged at the speed of 10,000 r/min for 10 min respectively, and the supernatant was discarded. Then, the precipitation with ultrapure water was dispersed using an ultrasonic method and centrifuged again. The samples were detected by SEM. When low concentration OTC was added, it combined with the Apt which was desorbed from the CuNCs to recover its catalytic activity, and a small amount of AuNPs were generated in lumps (Figure 2B). With the increasing concentration of OTC, more and more AuNPs were generated, with the size increasing (Figure 2C).

### 3.3. RRS Spectra of CuNCs, Apt Inhibition and OTC Analysis System

**Figure 3:**
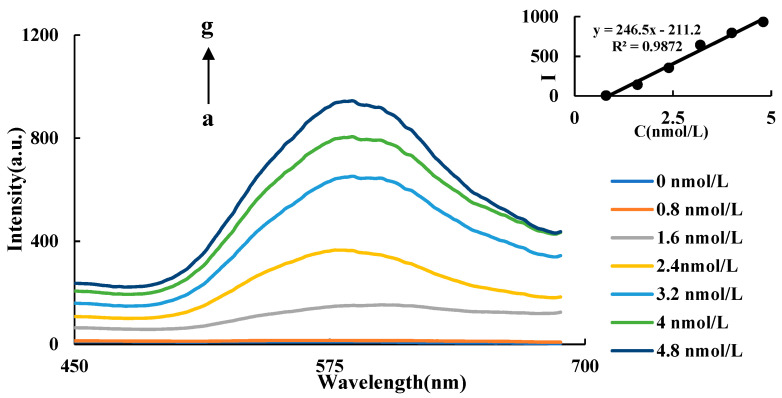
RRS spectra of CuNCs catalysis a: 4.2 μmol/LHAuCl4 + 3.5 mol/L ethanol + 0.5 mol/LHCl; b: a + 0.8 nmol/L CuNCs; c: a + 1.6 nmol/L CuNCs; d: a + 2.4 nmol/L CuNCs; e: a + 3.2 nmol/L CuNCs; f: a + 4 nmol/L CuNCs; g: a + 4.8 nmol/L CuNCs.

HAuCl_4_ can be reduced by ethanol to form AuNPs at above 85 °C, which is difficult to be performed at lower temperatures. CuNCs can catalyze this reaction at lower temperatures. In a certain range, with the increase of CuNCs, the catalytic effect was enhanced, and the number of AuNPs generated by CuNCs-HAuCl_4_-ethanol-HCl system increased gradually. In Figure 3, as the CuNCs concentration increased, the maximum peak is weakly blue shifted (from 590 nm to 582 nm). This is because, with the enhancement of catalytic effect, both the generation rate of the AuNPs and the nucleation rate were accelerated, resulting in the generation of more AuNPs with a small particle size. Therefore, the enhancement of the RRS peak is accompanied by a weak blue shift of the maximum peak while the CuNCs’ concentration increases. The RRS signal of the system showed an upward trend, and the maximum RRS peak was at 586 nm. As the concentration of the CuNCs increases, the value of *I*_586 nm_ of the system gradually increases. There is a good linear relationship between the concentration of CuNCs and the intensity of RRS in the range of 0.8–4.8 nmol/L (Figure 3). The linear equation is ∆*I*_586 nm_ = 246.5C − 211.2, and the linear correlation coefficient was 0.9872.

**Figure 4:**
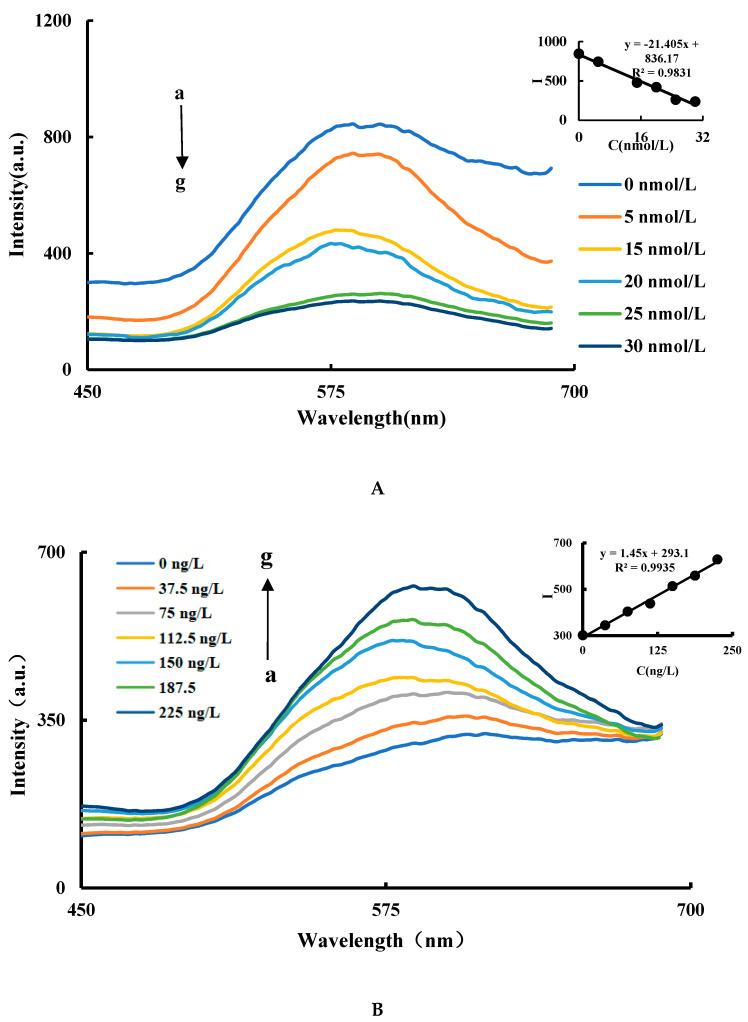
RRS spectra of Apt inhibition and the OTC analysis system (**A**) RRS spectra of Apt inhibition: a: 1.2 nmol/L CuNCs + 2.52 μmol/L HAuCl4 + 1.87 mol/L ethanol + 0.65 mol/LHCl; b: a + 5 nmol/L Apt; c: a + 10 nmol/L Apt; d: a + 15 nmol/L Apt; e: a + 20 nmol/L Apt; f: a + 25 nmol/L Apt; g: a + 30 nmol/L Apt. (**B**) RRS spectra of the OTC analysis system: a: 1.2 nmol/L CuNCs + 27.5 nmol/L Apt + 2.52 μmol/L HAuCl4 + 1.87 mol/L ethanol + 0.65 mol/L HCl; b: a + 37.5 ng/L OTC; c: a + 75 ng/L OTC; d: a + 112.5 ng/L OTC; e: a +150 ng/L OTC; f: a + 187.5 ng/L OTC; g: a + 225 ng/L OTC.

In the CuNCs-Apt-OTC-HAuCl_4_-ethanol-HCl system, the Apt can combine with the CuNCs to form a CuNCs-Apt complex due to electrostatic attraction. The catalytic activity of the CuNCs is inhibited, and the RRS signal decreases as the concentration of the Apt increases. In Figure 4A, with the enhanced inhibition of the aptamer, the reaction speed of the AuNPs generation slowed down and the product decreased, so the maximum peak decreased with a slight red shift. The optimal inhibition effect is achieved when the concentration of Apt is 25 nmol/L (Figure 4A). In the CuNCs-Apt-OTC-HAuCl_4_-ethanol-HCl-OTC system, with the addition of OTC, Apt in the system specifically combined with OTC, resulting in the release of CuNCs and the recovery of catalytic performance. In Figure 4B, with the increase of the OTC concentration, the maximum peak starts with a slight blue shift and then a slight red shift. That is probably because with the OTC addition, the catalytic activity of the CuNCs recovers, nucleation speed is accelerated, and more AuNPs are generated with smaller particle size. As the OTC concentration continues to increase, the nucleation rate does not accelerate, but the catalytic generation of AuNPs continues to increase, resulting in larger particle size and red shift of the maximum peak. In a certain concentration range, the RRS signal of the system increases linearly with the increase of the OTC concentration, and the maximum RRS peak is at 586 nm (Figure 4B). Therefore, the intensity at 586 nm of RRS signal and OTC concentration were selected as the working curve of mutual change relationship. As shown in Fig. 4B, the OTC concentration in the range of 37.5–225 ng/L has a good linear relationship with the intensity of RRS (∆*I*_586 nm_). The linear equation is ∆*I*_586 nm_ = 1.45C + 293.1, and the linear correlation coefficient is 0.9935.

### 3.4. SERS Spectra of OTC Analysis System

**Figure 5:**
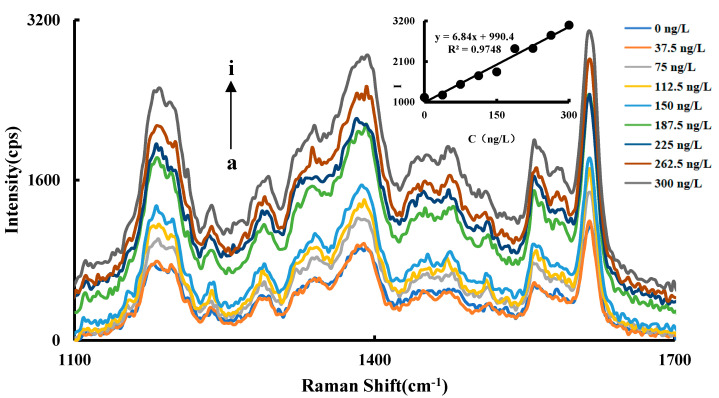
SERS spectra of the OTC analysis system a: 1.2 nmol/L CuNCs + 27.5 nmol/L Apt + 2.52 μmol/L HAuCl4 + 1.87 mol/L ethanol + 0.65 mol/L HCl; b: a + 37.5 ng/L OTC; c: a + 75 ng/L OTC; d: a + 112.5 ng/L OTC; e: a + 150 ng/L OTC; f: a + 187.5 ng/L OTC; g: a + 225 ng/L OTC; h: a + 262.5 ng/L OTC; i: a + 300 ng/L OTC.

The Raman signal of CuNCs-Apt-OTC-HAuCl_4_-ethanol-HCl-VB4R system increases linearly with the increase of OTC concentration in a certain concentration range, which has a maximum value at 1615 cm^−1^. Therefore, the relationship between the Raman signal strength at 1615 cm^−1^ and the change of the OTC concentration was selected as the working curve. The results showed that an OTC concentration in the range of 37.5–300 ng/L has a good linear relationship with SERS intensity ∆*I*_1615 cm_^−1^, and the linear equation is ∆*I*_1615 cm_^−1^ = 6.84C + 990.4, with a coefficient of 0.9748 (Figure 5).

### 3.5. Fluorescence and Stability of CuNCs

At a voltage of 500 V and a slit of 5 nm, the fluorescence characteristics of 0.04 mmoL/L CuNCs were investigated. In the system of CuNCs-Apt-OTC-HAuCl_4_-ethanol-HCl, only the CuNCs showed fluorescence characteristics. The fluorescence spectra of the CuNCs were detected with the excitation wavelength range of 350–400 nm. As the excitation wavelength increased, the maximum emission fluorescence peak shifted from 435 nm to 480 nm, and the fluorescence peak increased first and then decreased (Figure S1). The maximum fluorescence peak was at 475 nm when the excitation wavelength was 390 nm. The fluorescence properties of the prepared CuNCs were similar to those of other reported literatures [38,39], indicating that the preparation was successful.

CuNCs in solution tend to aggregate, precipitate, and lose catalytic activity over time. The fluorescence signals of the newly prepared CuNCs were measured continuously with time. The results show that the CuNCs were stable within one week.

### 3.6. Optimization of Experimental Conditions

The optimized reaction conditions of CuNCs-Apt-OTC-HAuCl_4_-ethanol-HCl system were studied according to the experimental method. When the concentration of CuNCs in the system was 1.2 nmol/L, Δ*I* had a maximum value (Figure S2A). When the Apt concentration in the system was 27.5 nmol/L, ΔI had a maximum value (Figure S2B). When the concentration of HAuCl_4_ in the system was 2.52 μmol/L, ΔI had the maximum value (Figure S2C). When the concentration of ethanol in the system was 1.87 mol/L, Δ*I* had the maximum value (Figure S2D). When the concentration of HCl in the system was 0.65 mol/L, Δ*I* had a maximum value (Figure S2E). When the heating temperature of the system was 60 °C, Δ*I* had a maximum value (Figure S2F). When the heating time of the system was 6 min, Δ*I* had the maximum value (Figure S2G). As such, the above optimized conditions were chosen for their use.

### 3.7. Working Curve

Under the optimized conditions, the working curves of RRS and SERS for the determination of the OTC were obtained (Table 1). The working curve of ∆*I*_586 nm_ = 1.45C + 293.1 was determined by RRS. The detection range of RRS is 37.5–225 ng/L with the LOD of 25.0 ng/L and the correlation coefficient of 0.9935. SERS has better sensitivity and a wider detection range, and the working curve is ∆*I*_1615 cm_^−1^ = 6.84C + 990.4. The detection range of SERS is 37.5–300 ng/L with the LOD of 18.0 ng/L and a coefficient of 0.9748. 

### 3.8. Comparison of Methods

The OTC analysis methods mainly include high performance liquid chromatography, electrochemical methods, fluorescence, surface plasmon resonance, SERS, and enzyme-linked immunosorbent assays. However, there are some defects in these methods. For example, according to the related references (Table S1), the HPLC method has a low sensitivity (μg/kg level), and the downside of fluorescence is that its material preparation is complex. Compared with some reported methods for the determination of OTC (Table S1), this method in our work has the advantages of simplicity, speed, sensitivity, and selectivity.

### 3.9. The Influence of Coexisting Substances

According to the requirements of the control variable method, the influence of coexisting substances in the determination of 150 ng/L OTC by CuNCs-Apt-OTC-HAuCl_4_-ethanol-HCl system was investigated. When the relative error was within ±10%, 100 times of Na^+^, Ca^2+^, Mg^2+^, K^+^, NH_4_^+^, penicillin sodium, penicillin potassium, tetracycline, ofloxacin, NO_3_^−^, SO_4_^2−^, bovine serum albumin (BSA), human serum albumin (HSA), doxycycline, vitamin B_12_, tryptophan, glycine, 50 times of PO_4_^3−^, chloramphenicol, and 10 times of Fe^3+^ did not interfere with the determination of OTC (Tables S2 and S3). The results showed that this method has a good selectivity.

### 3.10. Determination of the Samples

Three different OTC samples, including A (0.25 g/tablet, Gansu Qilian Mountain Pharmaceutical Co., Ltd., Jiuquan, China), B (0.25 g/tablet, Teyi Pharmaceutical Group Co., Ltd., Taishan, China) and C (0.125 g/tablet, Anhui Golden Sun Biochemical Pharmaceutical Co., Ltd., Fuyang, China) were taken, then dissolved after grinding and diluted to a certain multiple. A 1.0 mL sample solution was accurately absorbed and added into the optimized CuNCs-Apt-OTC-HAuCl_4_-ethanol-HCl system, and 5 parallel measurements were performed by the RRS method for each sample. The determination results are as follows (Table 2). Relative standard deviation (RSD) can reflect the precision of the test results. The recovery is an index reflecting the degree of loss of the analytes in the sample analysis process. The less the loss, the higher the recovery rate. It has a close relationship with the accuracy and the stability of the analysis. The relative standard deviations are between 3.8 and 5.2%, and the recoveries are between 97.13 and 103.4%. That means the new assay is accurate, sensitive, and stable.

## 4. Conclusions

In this work, CuNCs with high catalytic activity were prepared using a simple method, and their morphology was characterized by SEM and TEM. The CuNCs were applied as catalysis to the reaction of HAuCl_4_-ethanol, and the product of AuNPs has a strong RRS/SERS signal. To realize the sensitive detection of OTC, the electrostatic attraction between the CuNCs and the Apt was used to regulate the catalytic properties of nanoclusters. This method has the advantages of simplicity, rapidity, stability, and sensitivity, and provides a new assay for OTC detection.

## Figures and Tables

**Table 1:** Working curve of RRS/SERS for measuring OTC.

Method	Linearity Range (ng/L)	Regression Equation	Coefficient	LOD (ng/L)
RRS	37.5–225	∆*I*_586 nm_ = 1.45C + 293.1	0.9935	25.0
SERS	37.5–300	∆*I*_1615 cm_^−1^ = 6.84C + 990.4	0.9748	18.0

**Table 2:** RRS results of water samples.

Sample	Detected Value (ng/L)	Average (ng/L)	Add (ng/L)	Found (ng/L)	Recovery (%)	RSD (%)	Content (g/Tablet)
A	46.13, 42.52, 45.56, 42.80, 43.30	44.06	150	201.0	103.4	3.8	0.2203
B	43.12, 48.50, 48.38, 45.06, 47.95	46.60	150	191.0	97.13	5.2	0.233
C	106.8, 113.4, 109.2, 114.9, 103.7	109.6	150	262.4	101.8	4.2	0.1096

## Data Availability

The raw/processed data required to reproduce these findings cannot be shared at this time as the data also forms part of an ongoing study.

## Supplementary Materials

The following are available online at https://www.mdpi.com/article/10.3390/nano11102501/s1, Figure S1: Fluorescence spectra of CuNCs with different excitation wavelengths, Figure S2A–G: Optimization experiments; Table S1: Comparison of characteristics of some OTC analysis methods reported, Table S2: Influence of interfering ions on RRS determination of OTC, Table S3: Influence of interfering ions on SERS determination of OTC.
