# Supplementary material for: Sensitive Aptamer SERS and RRS Assays for Trace Oxytetracycline Based on the Catalytic Amplification of CuNCs
Source: Nanomaterials (Basel). 2021 Sep 26;11(10):2501. doi: 10.3390/nano11102501 (PMC8541458; doi:10.3390/nano11102501)
Supplement: Supplementary file 1 [file nanomaterials-11-02501-s001.zip › nanomaterials-1366347-supplementary.pdf]

nanomaterials

Article

Sensitive Aptamer SERS and RRS Assays for Trace Oxytetracycline Based on the Catalytic Amplification of CuNCs

Shuxin Chen [1,2], Xiaowen Lv [1,2], Jifan Shen [1,2], Siqi Pan [1,2], Zhiliang Jiang [1,2], Yang Xiao [1,2] and Guiqing Wen [1,2,*]

1 Key Laboratory of Ecology of Rare and Endangered Species and Environmental Protection (Guangxi Normal University), Ministry of Education, Guilin 541004, China; shuxinchen666@163.com (S.C.); xwlv9785178@163.com (X.L.); ddyeah88@163.com (J.S.); sqpan1130@126.com (S.P.); zljiang@gxnu.edu.cn (Z.J.); xy1169311611@163.com (Y.X.)

2 Guangxi Key Laboratory of Environmental Pollution Control Theory and Technology for Science and Education Combined with Science and Technology Innovation Base, Guilin 541004, China

* Correspondence: gqwen@gxnu.edu.cn

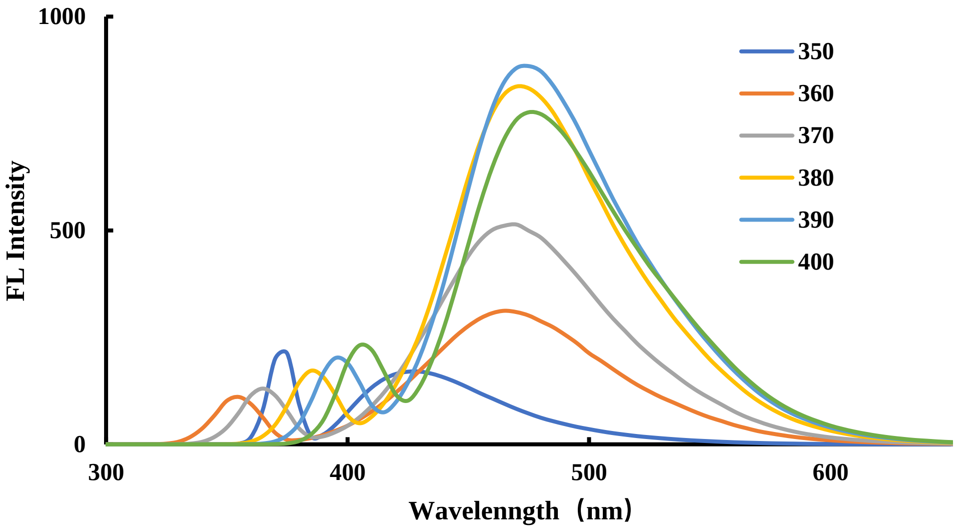

Figure S1. Fluorescence spectra of CuNCs with different excitation wavelengths 0.4 mmol/L CuNCs+27.5 nmol/L Apt+2.52 μmol/L HAuCl4+1.87 mol/L ethanol +0.65 mol/L HCl

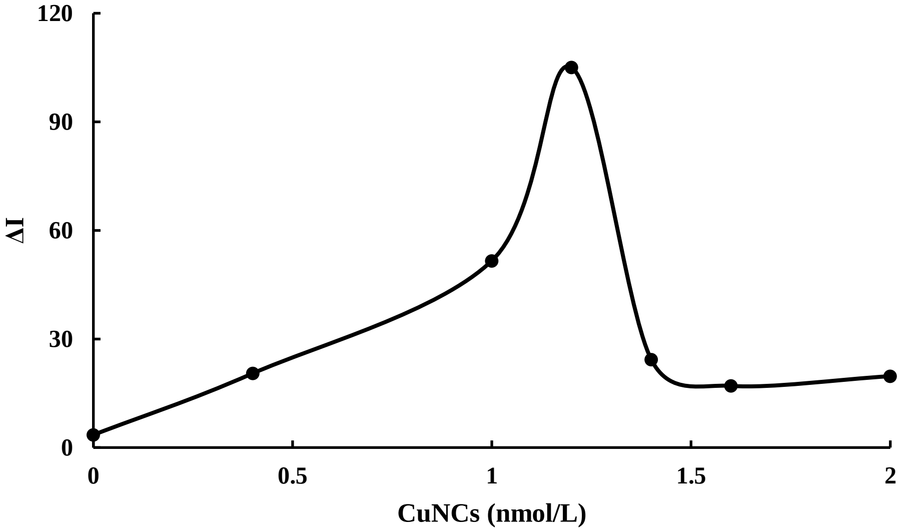

Figure S2. A. Optimization of CuNCs concentration CuNCs+ 25 nmol/L Apt+75 ng/L OTC+4.2 μmol/L HAuCl4+3.5 mol/L ethanol +0.5 mol/LHCl.

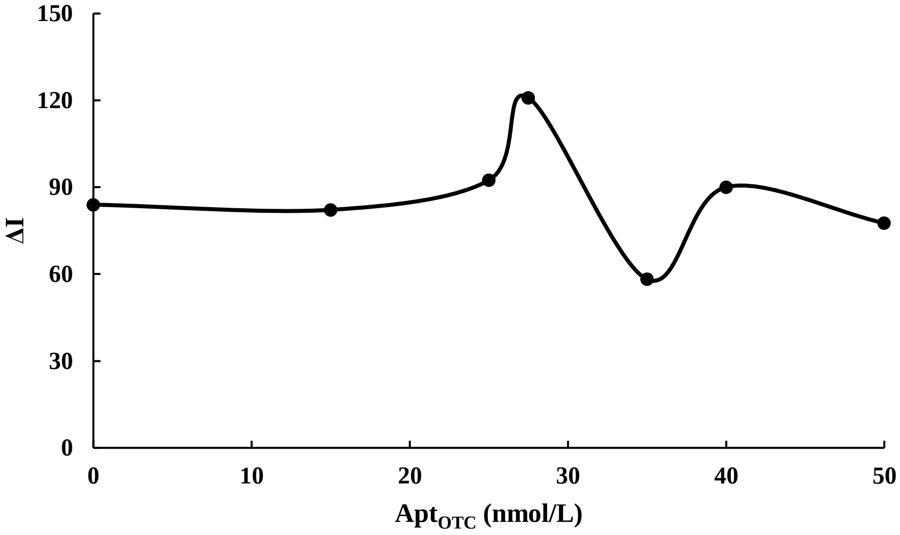

Figure S2. B. Optimization of Apt concentration 1.2 nmol/L CuNCs+Apt+75 ng/L OTC+4.2 μmol/L HAuCl4+3.5 mol/L ethanol +0.5 mol/LHCl.

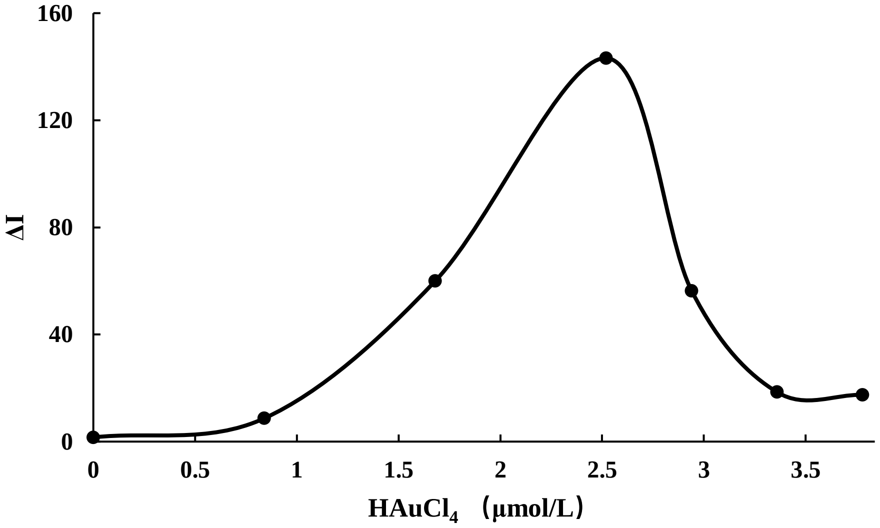

Figure S2. C. Optimization of HAuCl4 concentration 1.2 nmol/L CuNCs+27.5 nmol/L Apt+75 ng/L OTC+HAuCl4+3.5 mol/L ethanol +0.5 mol/LHCl.

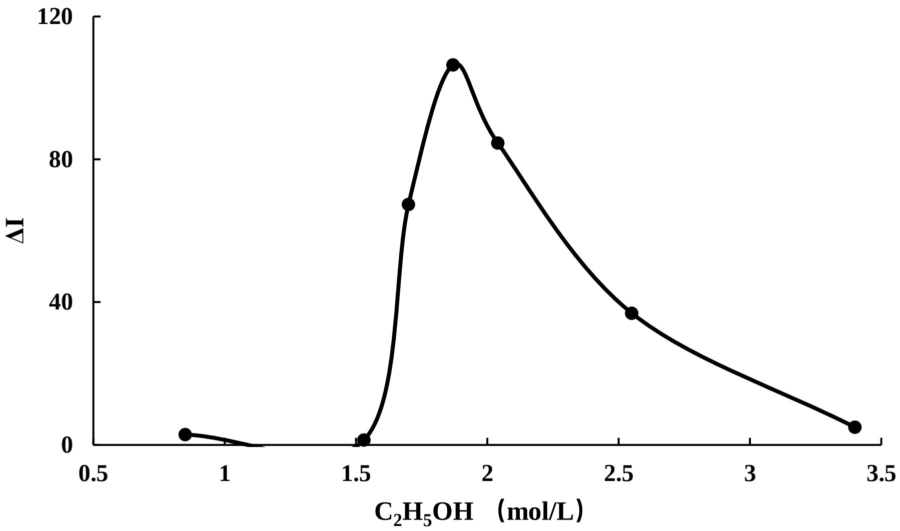

Figure S2. D. Optimization of HAuCl4 concentration 1.2 nmol/L CuNCs+27.5 nmol/LApt+75ng/LOTC+2.52μmol/L HAuCl4+ethanol+0.5 mol/LHCl.

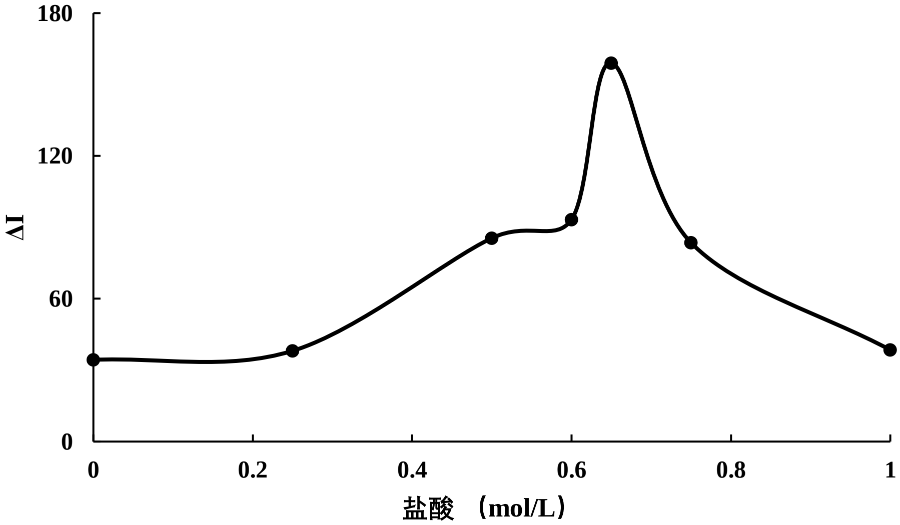

Figure S2. E. Optimization of HCl concentration 1.2 nmol/L CuNCs+27.5 nmol/L Apt+75 ng/L OTC+2.52 μmol/L HAuCl4+1.87 mol/L ethanol +HCl.

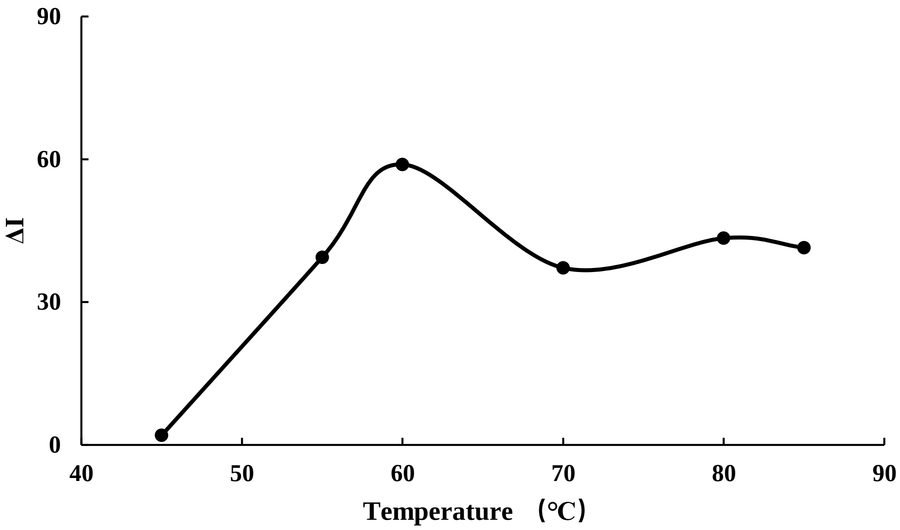

Figure S2. F. Temperature optimization 1.2 nmol/L CuNCs+27.5 nmol/L Apt+75 ng/L OTC+2.52 μmol/L HAuCl4+1.87 mol/L ethanol +0.65 mol/LHCl.

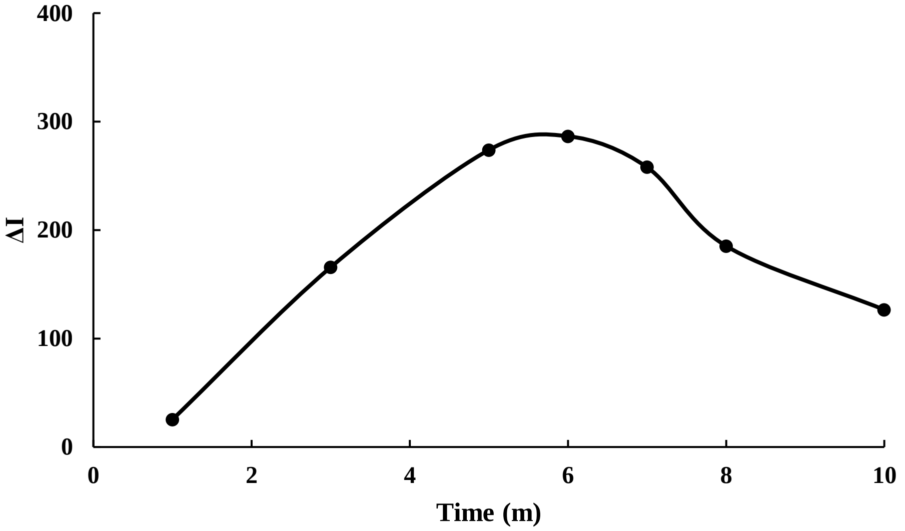

Figure S2. G. Time optimization 1.2 nmol/L CuNCs+27.5 nmol/L Apt+75 ng/L OTC+2.52 μmol/L HAuCl4+1.87 mol/L ethanol +0.65 mol/LHCl.

Table S1. Comparison of characteristics of some OTC analysis methods reported.

method	principle	Linear range	LOD	Characteristic analysis	Ref.
High performance liquid chromatography (HPLC)	The samples of OTC were extracted by liquid-liquid extraction and solid phase extraction, and separated and quantified by high performance liquid chromatography and fluorescence	100–350μg /kg	10μg /kg	It can be used for simultaneous determination of multiple antibiotics	[30]
Electrochemical method	A visible-light-driven photoelectrochemical (PEC) OTC aptasensor was constructed using Bi4VO8Cl/nitrogen-doped graphene quantum dots (Bi_4VO_8Cl/N-GQDs) nanohybrids as photoactive material and OTC aptamer as identification element. Detect OTC a ccording to changes in electrochemical signal.	0.1–150 nmol/L	0.03 nmol/L	Good precision, accurate and reliable	[31]
Fluorescence	In the presence of Fe3O4 magnetic nanoparticles and H_2O_2, the fluorescence of CDs can be quenched by OTC, so an OTC detection method is established.	25nmol/L–1.75μmol/L	9.5 nmol/L	It has the advantages of high accuracy and simple sample pretreatment ,but The downside is that complex material preparation.	[32]
Surface plasmon resonance(SPR)	Based on the catalytic amplification method, LCNPs can catalyze NaH_2PO_2-$HAuCl_4$ to generate gold nanoparticles, which changes the SPR signal. The aptamer can interact with LCNPs to form a complex, which	1.0–7.5 ng/mL	0.5 ng/mL	Novel, high sensitivity, simple operation and low cost	[33]

	inhibits the catalytic effect of LCNPs, while OTC can specifically bind with the aptamer to release LCNPs and catalyze recovery.				
SERS	The DNA sequence combined with the OTC aptamer and Raman signal molecule (4-MBA) were used to modify the gold nanoparticles. In the presence of OTC, the aptamer combined with the surface of the gold nanoparticles and the Raman scattering signal was enhanced.	4.60×10^{-2} – 4.60×10^{2} fg/mL	4.35×10^{-3} fg/mL	Wide linear range and low detection limit	[34]
Enzyme-linked immunosorbent assay (ELISA)	A kind of disposable porous three-layer filter membrane was prepared, and the rapid multi-silver enhancement was achieved by filtering and capturing OTC through the specific recognition substance in each layer	2–128.46 ng/mL	2 ng/mL	Large linear range, simple operation	[35]
This method	CuNCs catalysed the HAuCl4-ethanol system with a high RRS signal. Apt can inhibit the reaction activity of CuNCs, which can regulate the inhibition degree of the aptamer. The oxytetracycline in the system can be detected by RRS	37.5–300 ng/L	18.0 ng/L	Stable, sensitive, simple operation, low cost	This method

Table S2. Influence of interfering ions on RRS determination of OTC.

Coexistence of material	Relative ratio	Relative error (%)	Coexistence of material	Relative ratio	Relative error (%)
Na^{+}	100	1.17	Ca^{2+}	100	−1.93
Mg^{2+}	100	4.81	K^{+}	100	−4.97
NH_4^{+}	100	2.4	penicillin sodium	100	−2.87
penicillin potassium	100	3.45	tetracycline	100	4.68
ofloxacin	100	−7.04	NO_3^{-}	100	−5.7
SO_4^{2-}	100	−0.42	BSA	100	−4.4
HSA	100	4.3	doxycycline	100	1.91
vitamin B_{12}	100	−0.52	tryptophan	100	−0.11
glycine	100	−0.86	PO_4^{3-}	50	−2.66
chlorampheni col	50	0.04	Fe^{3+}	10	9.82

Table S3. Influence of interfering ions on SERS determination of OTC.

Coexistence of material	Relative ratio	Relative error (%)	Coexistence of material	Relative ratio	Relative error (%)
Na^{+}	100	0	Ca^{2+}	100	−9.15
Mg^{2+}	100	1.65	K^{+}	100	5.86
NH_4^{+}	100	3.84	penicillin sodium	100	1.14
penicillin potassium	100	4.13	tetracycline	100	9.63
ofloxacin	100	−0.17	NO_3^{-}	100	−3.29
SO_4^{2-}	100	6.47	BSA	100	3.13
HSA	100	−6.77	doxycycline	100	4.21
vitamin B_{12}	100	2.37	tryptophan	100	−0.92
glycine	100	−1.98	PO_4^{3-}	50	−3.3
chlorampheni col	50	1.9	Fe^{3+}	10	9.63
